# *In silico* investigation of heparanase-correlated genes in breast cancer subtypes

**DOI:** 10.31744/einstein_journal/2020AO5447

**Published:** 2020-10-02

**Authors:** Carina Mucciolo Melo, Henrique Pereira Prado, Gabriela Araújo Attie, Daniel Lacaz Ruiz, Manoel João Batista Castello Girão, Maria Aparecida da Silva Pinhal

**Affiliations:** 1 Faculdade de Medicina do ABC Santo AndréSP Brazil Faculdade de Medicina do ABC, Santo André, SP, Brazil.; 2 Universidade Federal de São Paulo São PauloSP Brazil Universidade Federal de São Paulo, São Paulo, SP, Brazil.; 3 McMaster University HamiltonON Canada McMaster University, Hamilton, ON, Canada.

**Keywords:** Heparanase-1, Computational biology, Breast neoplasms

## Abstract

**Objective:**

To investigate the possible genes that may be related to the mechanisms that modulate heparanase-1.

**Methods:**

The analysis was conducted at *Universidade Federal de São Paulo*, on the data provided by: The Cancer Genome Atlas, University of California Santa Cruz Genome Browser, Kyoto Encyclopedia of Genes and Genomes Pathway Database, Database for Annotation, Visualization and Integrated Discovery Bioinformatics Database and the softwares cBioPortal and Ingenuity Pathway Analysis.

**Results:**

Using messenger RNA expression pattern of different molecular subtypes of breast cancer, we proposed that heparinase-1 was co-related with its progression. In addition, genes that were analyzed presented co-expression with heparanase-1. The results that showed that heparanase-1 co-expressed with phosphoinositide 3-kinase adapter protein 1, sialic acid-binding immunoglobulin-like lectin 7, and leukocyte-associated immunoglobulin-like receptor 1 are directed related with immune system evasion during breast cancer progression. Furthermore, cathepsin L was co-expressed with heparanase-1 and transformed inactive heparanase-1 form into active heparanase-1, triggering extracellular matrix remodeling, which contributes to enhanced tumor-host interaction of the tumor.

**Conclusion:**

The signaling pathway analysis using bioinformatics tools gives supporting evidence of possible mechanisms related to breast cancer development. Evasion genes of the immune system co-expressed with heparanase-1, a enzyme related with tumor progression.

## INTRODUCTION

The publication of the first draft of the human genome in 2001 marked the beginning of a new era in biology: the age of bioinformatics. Given the sheer amount of information being digitalized, genomics data can only be clearly understood with the help of data science and bioinformatics tools, such as pathway analysis software. Since the rate of these databases grow exponentially, dependence on the part of health professionals and researchers to these computational tools seem to be ever increasing.^(1)^ Although database analysis on widely available virtual databases are a useful resource, accessibility to use bioinformatics data has not been widely spread throughout the scientific community, due to the expertise necessary to use databanks.

One of the objectives of this study is to provide guiding documentation to healthcare professionals on how to answer biological questions relating to specific gene expression, and its correlation with other signaling pathways.

We propose to conduct this study on both isoforms of heparanase, namely heparanase-1 (HPSE) and heparanase-2 (HPSE2), in the context of breast cancer.

Heparanase-1 is an endo-beta-glucuronidase that participates in the cleavage of heparan sulfate and heparin chains. The oligosaccharides generated by the enzymatic action of HPSE interact with cytokines, angiogenic factors and growth factors, thus activating processes related to the tumor progression. In addition, recent studies showed the correlation of HPSE with exosome formation, activation of the immune system, autophagy, and chemoresistance, demonstrating its importance in modulation of the crosstalk between tumor cells and the tumor environment. As a result of this finding, HPSE is a target for development of new drugs for cancer, and anti-HPSE treatment can be used to stimulate T cells and initiate anti-cancer immune responses.^(2,3)^

Unlike HPSE, HPSE2 does have a catalytic property, and its function is still very cryptic. However, it is well known that HPSE2 interacts with heparin and heparan sulfate, therefore it could be used to hamper the effects of HPSE.^(4)^

Despite the importance of HPSE and its contribution to carcinogenesis, angiogenesis, and inflammation, there is a limited amount of work detailing how other genes might be correlated with HPSE’s mechanisms of action. To answer this question, we used genomics databases freely available online as a source of analysis.

Furthermore, correlation of HPSE and HPSE2 in different human breast cancers was addressed to answer if both heparanases could be a useful marker to differentiate among such subtypes of cancer.

## OBJECTIVE

To investigate the possible genes that may be related to the mechanisms that modulate heparanase-1.

## METHODS

### Genomic databases

Analysis was conducted at *Universidade Federal de São Paulo*, *São Paulo (*SP), Brazil, on data provided by the The Cancer Genome Atlas (TCGA), University of California Santa Cruz, Genome Browser, Kyoto Encyclopedia of Genes and Genomes (KEGG) Pathway Database, Database for Annotation, Visualization and Integrated Discovery (DAVID) and Bioinformatics Database. The analysis was performed between 2017 and 2019. This study did not require Ethics Committee approval.

### Cloud services

The web-based cloud services were used to analyze genomics databases: cBioPortal web-based application for TCGA database analysis.

### Cohort data collection

Data was collected from breast cancer patients as part of the TCGA invasive breast carcinoma project, and classified according to cancer sub-type: luminal A, luminal B, human epidermal growth factor receptor-type 2 (HER2) positive and triple negative.

### Correlation analysis of heparanase and co-expressed genes

cBioPortal cloud services relating to genomic databases were used to analyze genetic expression from the selected cohort.

### Signaling pathway analysis

The pathways of co-expressed genes with the isoforms of HPSE were analyzed Ingenuity Pathway Analysis (IPA) software.

### Statistical significance test

The software SPSS (SPSS, Illinois, USA), version 17, was used to analyze the dataset provided by the genomic databases, while using a statistical significance threshold of 5% (p≤0.05). All datasets were imported after being reorganized in tables. Kolmogorov-Smirnov and Student’s *t* test were both applied by this software.

## RESULTS

**Figure 1:**
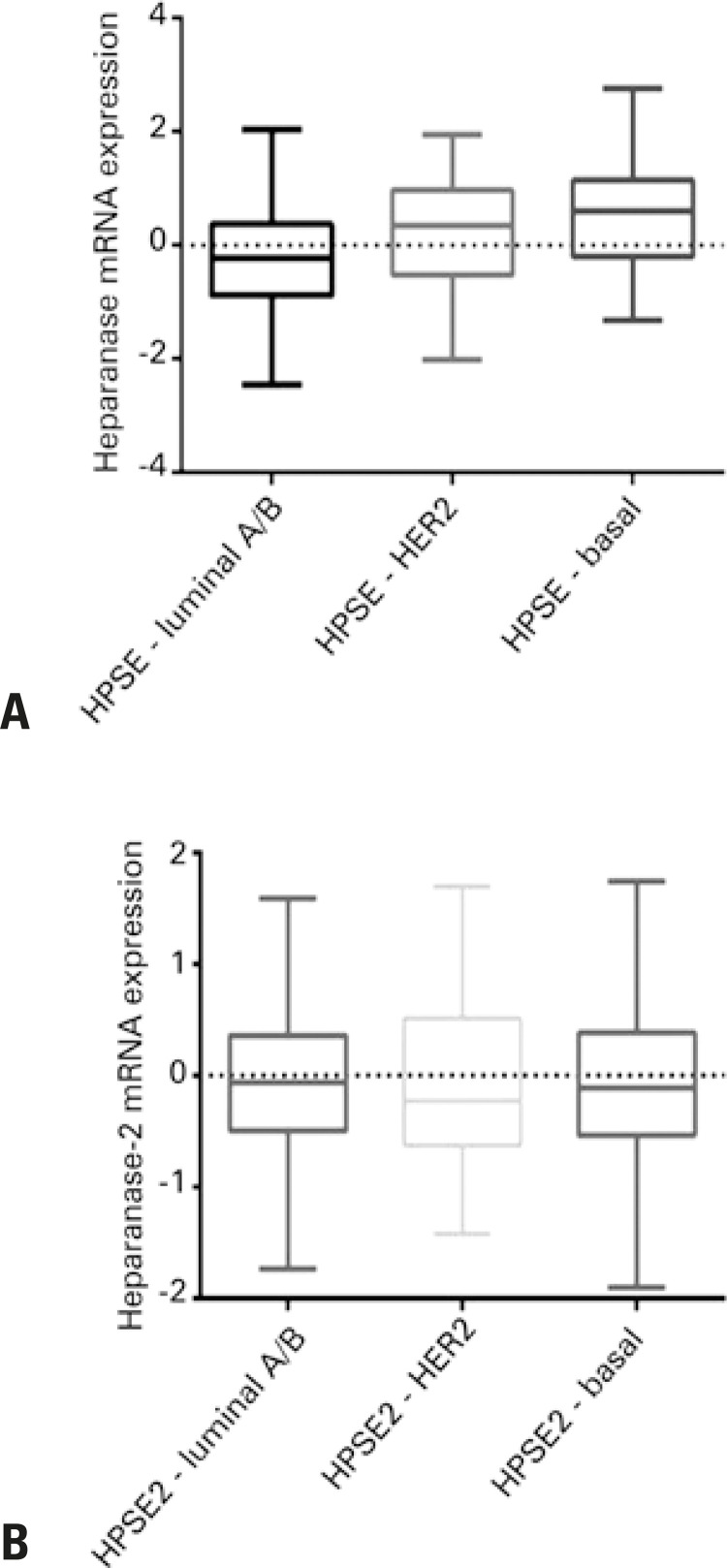
Heparanase-1 and heparanase-2 messenger RNA expression in different subtypes of breast cancer. Relative expression of (A) heparanase-1 and (B) heparanase-2 messenger RNA, in luminal A/B (n=321), human epidermal growth factor receptor 2 enriched (n=58), and basal/triple-negative (n=81) breast cancer subtypes. Data was obtained from The Cancer Genome Atlas Breast Carcinoma database (2012).

Initially, we accessed www.cbioportal.org and downloaded data pertaining to invasive breast carcinoma (TCGA, Nature 2012).^(5,6)^ The collected data refer to the expression of messenger RNA (mRNA) for HPSE and HPSE2 in different subtypes of breast cancer, such as luminal A/B (n=321), triple-negative/basal (n=81), and HER2 (n=58) (Figure 1).

mRNA: messenger RNA; HPSE: heparanase-1; HER2: human epidermal growth factor receptor-type 2; HPSE2: heparanase-2.

There were a significant statistical difference between HPSE expressions in various breast cancer subtypes. However, no difference was detected for HPSE2.

Granted HPSE2 showed no significant correlation among tumor subtypes, we used HPSE to evaluate co-expressed genes. The analysis of the TCGA database has shown genes that positively correlate with HPSE expression. This includes phosphoinositide-3-kinase adaptor protein 1 (PIK3AP1), sialic acid-binding immunoglobulin-like lectin 7 (SIGLEC7), leukocyte-associated immunoglobulin-like receptor 1 (LAIR1) and cathepsin L (CTSL), which we have selected for further study based on their co-expression with HPSE.

Phosphoinositide-3-kinase adaptor protein 1 plays a multitude of roles in immune response, and has a crucial function as a downstream component of several immune-related receptors, in various cell types. In B-cells, PIK3AP1 links the B-cell receptor to the p85 subunit of phosphoinositide-3-kinase (PI3K), and is responsible for cell maturation, while in natural killer (NK) cells this protein acts as a mediator between NK inhibitory receptors and PI3K/protein kinase B alpha (AKT).^(7-9)^

**Figure 2:**
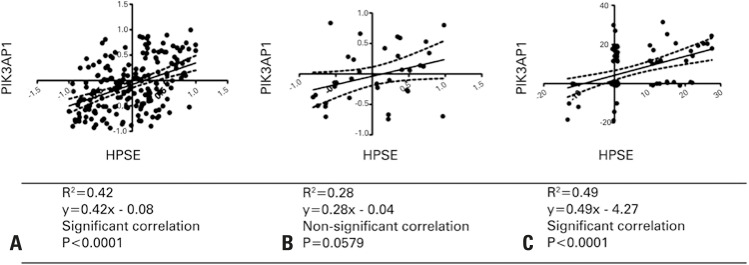
Heparanase-1 and adaptor for phosphoinositide 3-kinase correlation in different subtypes of breast cancer. (A) luminal A/B (n=321) (B) enriched (n=58) and (C) triple negative (n=81)

PIK3AP1: phosphoinositide-3-kinase adaptor protein 1; HPSE: heparanase-1.

Figure 2 shows a significant correlation between the increase of HPSE expression and the expression of PIK3AP1 in luminal A/B and triple-negative tumor subtypes. However, we did not observe a meaningful correlation between HPSE and PIK3AP1 in HER2-enriched tumors, indicated by a non-significant R^2^ coefficient and p value.

**Figure 3:**
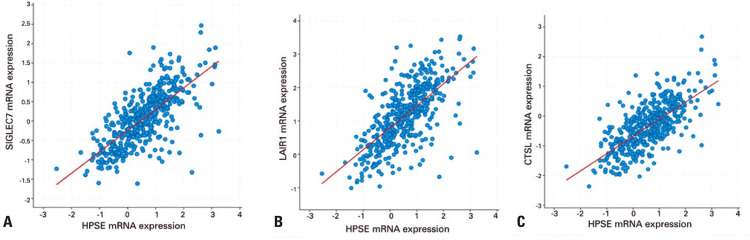
Messenger RNA co-expression with heparanase-1. (A) Expression of sialic-acid-binding immunoglobulin-like lectin-7 in relation to messenger RNA expression of heparanase-1. Data points denote breast cancer patient. Regression line is modelled by y=0.55x-0.24, R2=0.51, p=3.06 x 10-76, n=460 (B) Messenger RNA expression of inhibitory leukocyte-associated immunoglobulin-like receptor in relation to messenger RNA expression of heparanase-1. Data points denote breast carcinoma patient. Regression line is modelled by y=0.66x+0.79, R2=0.46, p=1.24 x 10-63, n=460. (C) Messenger RNA expression of cathepsin L in relation to messenger RNA expression of heparanase-1. Data points denote breast cancer patients. Regression line is modelled by y=0.58x-0.69, R2=0.48, p=6.17 x 10-65, n=460

SIGLEC7: sialic-acid-binding immunoglobulin-like lectin-7; HPSE: heparanase-1; mRNA: messenger RNA; LAIR1: inhibitory leukocyte-associated immunoglobulin-like receptor; CTSL: cathepsin L.

Sialic-acid-binding immunoglobulin-like lectin-7 is an inhibitory receptor found on NK cells. Once bound by sialic acid, SIGLEC7 causes a downstream cascade that prevents degranulation of NK cells.^(10)^ A significant positive correlation between HPSE and SIGLEC7 was observed in luminal A/B, HER2-enriched, and triple-negative breast cancer (y=0.55x-0.24, R^2^=0.51, p=3.06 x 10^-76^, n=460; basal subtype, y=0.1191x+0.5550, R^2^=0.52, p=0.0003; HER2-enriched, y=0.1387x+0.6429, R^2^=0.6509, p<0.0001; Luminal, y=0.003224x+0.4915, R^2^ =0.5019, p<0.0001) (Figure 3A). Interestingly, it is important to emphasize that tumor cells overexpress sialydated ligands, which results in an increased inhibition of immune responses.^(11)^

Inhibitory LAIR1 is a transmembrane glycoprotein that is a receptor to collagen which promotes suppression of immune responses. Leukocyte-associated immunoglobulin-like receptor 1 is expressed on most immune cells, including NK cells, T- and B-lymphocytes, eosinophils, and basophils.^(12)^

There was a direct correlation between LAIR1 and HPSE expression (y=0.66x+0.79, R^2^=0.46, p=1.24 x 10^-63^, n=460; basal subtype, y=0.5386x-0.08710, R^2^=0.5204, p=0.0014; HER2-enriched, y=0.6002x+0.09642, R^2^=0.5918, p=0.0003; Luminal, y=0.4148x+0.02971, R^2^=0.4061, p<0.0001), as shown in figure 3B. Cathepsin L is a cysteine protease,^(13)^ which shows a positive correlation with HPSE in three breast carcinoma subtypes, luminal A/B, HER2-enriched, and triple-negative (y=0.58x-0.69, R^2^=0.48, p=6.17 x 10^-65^, n=460; basal subtype, y=0.4291x-0.005506, R^2^=0.4691, p=0.0034; HER2-enriched, y=0.3187x-0.1288, R^2^=0.3480, p=0.0437; Luminal, y=0.3936x+0.003278, R^2^ =0.4144, p<0.0001) (Figure 3C).

Analysis of the TCGA database showed that the expression of genes HPSE, PIK3AP1, SIGLEC7, LAIR1, and CTSL have positive correlations according to the breast cancer subtypes. While HPSE and CTSL are involved in extracellular matrix (ECM) remodeling, PIK3AP1, SIGLEC7, and LAIR1 are part of signaling pathways involved with inhibition of immune system.^(12-15)^

## DISCUSSION

Great progress has been made in the past years to shine some light on mechanisms for immune evasion and carcinogenesis. The basic concepts of this process involve immune cells presenting receptors capable of preventing an inflammatory exacerbation that occurs during tumor development. The same mechanisms that prevent autoimmune disorders can also be used to decrease anti-tumor responses.^(15)^

The signaling pathway mediated by PI3K promotes the phosphorylation of AKT, resulting in a crucial event in tumorigenesis. Active AKT promotes the localization of forkhead box O1 (FOXO), β-catenin, among other factors to the cytoplasm, thus modulating mechanisms relating to cell proliferation, apoptosis, and metastasis.^(14-17)^

B cell adaptor for PIK3AP1, also named BCAP, is a signaling adaptor that activates the PI3K pathway downstream of cell receptor signaling in B cells and Toll-like receptor (TLR) signaling in macrophages. Phosphoinositide-3-kinase adaptor protein 1 binds to the regulatory p85 subunit of class I PI3K and is a large multi-domain protein.^(16)^

Phosphoinositide-3-kinase adaptor protein 1 is involved in multiple receptor responses, and confers varying responses depending on cell type. The expression of PIK3AP1 involves the activation of TLR which have important rolls in the inflammatory process, regulation of the immune response, and in cellular proliferation. Activated TLR promote the activation of the PIK3AP1 pathway, which leads to the production of nuclear factor kappa B (NF-κB), thus regulating cytokine expression through various complexes, including MyD88, TIRAP/Mai and TRF. Activation of the NF-κB pathway solicits a response from the adaptive immune system, through the production of inflammatory cytokines, such as interleukin (IL) 1, IL-8, and tumor necrosis factor alpha (TNF-α), among other pro-inflammatory factors, especially in circulating cells, such as NK cells and macrophages. Nuclear factor kappa B has a fundamental role in the regulation of the immune response during a bacterial or viral infection. The incorrect regulation of NF-κB leads to tumor development, inflammatory diseases, and autoimmune conditions.^(14,17,18)^

The SIGLEC7 receptor, present on NK cells and macrophages, binds to alternating glycol-conjugates on the surface of tumor cells. Such interaction prevents the NK cells from provoking the death of tumor cells through degranulation.^(19)^ Interestingly, an increased expression of SIGLEC7 was observed in macrophages that reside in the tumor microenvironment.^(19,20)^ Sialic acid-binding immunoglobulin-like lectin 7 present on monocytes induce the production of inflammatory factors, such as TNF-α, IL-1α, IL-6, IL-8, MIP-1β.^(10)^ Furthermore, pathogens and tumor cells that overexpress SA and carbohydrate ligands of SIGLEC7 contribute to the inhibition of NK and T cells. Therefore, SIGLEC7 seem to be related with tumor escape.^(19)^

The LAIR1 receptor interacts with collagen found in the ECM of tumors, thus inhibiting the cytotoxicity of NK and T-lymphocytes. Also, tissues obtained from human cervical cancer present higher expression comparing to normal tissues, as verified in HPSE expression.^(12,21,22)^

The CTSL cleaves multiple ECM components, such as fibronectin, collagen, and laminin. It is also known that CTSL processes pro-HPSE (inactive) into HPSE (active), once both protein complexes have been secreted into the extracellular milieu, and is super-expressed in various types of tumors, such as glioma, melanoma, and liver, breast and prostate cancer, and activates HPSE through a proteolytic mechanism. HPSE and CTSL are both important factors in the remodeling of the ECM.^(13)^Zhang et al., verified that CTSL and HPSE were increased in ovarian cancer and both were correlated to tumor progression.^(23)^

Cathepsin L seems to have important roles in tumorigenesis in both the nucleus and in the ECM. Secreted CTSL is capable of degrading ECM components, such as laminin, collagen type I and IV, fibronectin, elastin, and E-cadherin, thus playing a major role in the remodeling of the ECM. Furthermore, CTSL participates in the proteolytic activation of urokinase plasminogen, and cathepsin D, which both act as tumorigenic factors.^(13,24,25)^ The CTSL in the nucleus is responsible for Cux p200 processing, which leads to the production of mesenchymal genes. It also degrades S38P1, which activates DNA repair.^(13)^

Other factors that may affect the tumor microenvironment are hypoxia and acidosis, which contribute to the secretion of CTSL from the lysosome and, therefore, increase the proteolytic degradation of ECM and the basal membrane, thus facilitating tumor metastasis.^(13)^

**Figure 4:**
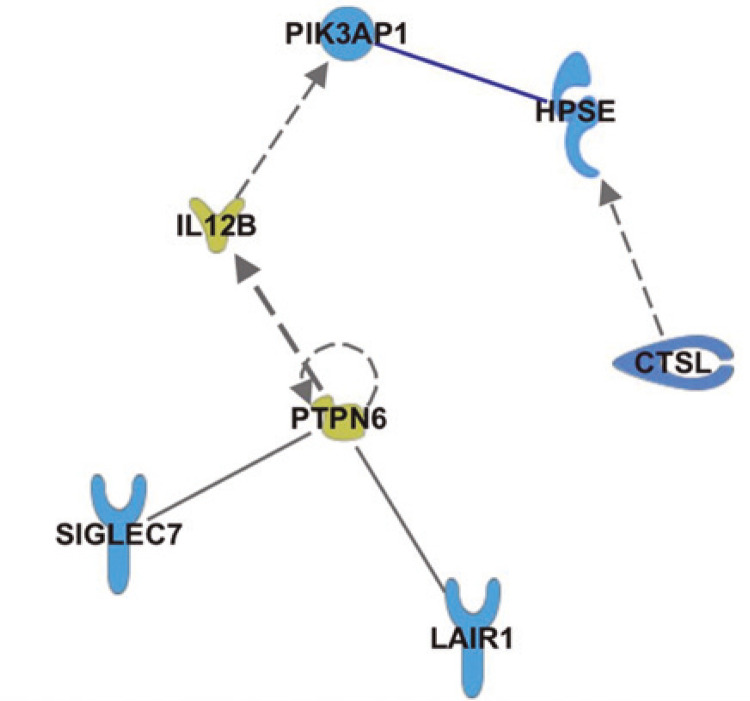
Schematic showing the relationship between heparanase, adaptor for phosphoinositide 3-kinase, cathepsin L, sialic-acid-binding immunoglobulin-like lectin-7 and inhibitory leukocyte-associated immunoglobulin-like receptor

HPSE: heparanase; PIK3AP1: adaptor for phosphoinositide 3-kinase; CTSL: cathepsin L; SIGLEC7: sialic-acid-binding immunoglobulin-like lectin-7; LAIR1: inhibitory leukocyte-associated immunoglobulin-like receptor.

Figure 4 shows possible protein interactions between HPSE, PIK3AP1, SIGLEC7, LAIR1 and CTSL, which we have inferred from pathway analysis using the software Ingenuity Pathway Analysis. Phosphoinositide-3-kinase adaptor protein and CTSL are directed related with HPSE. Phosphoinositide-3-kinase/AKT signaling pathways triggers HPSE expression, and induce epithelial to mesenchymal transition signaling metastasis of hepatocarcinoma.^(26)^ Cathepsin L is involved in the activation of HPSE and both proteins actively participate in the process of tumor development.^(27)^ Moreover, LAIR1 and SIGLEC7 correlate with HPSE, and interaction between SIGLEC7 and HPSE seems to be mediated by tyrosine-protein phosphatase non-receptor type 6 (PTPN6) and IL-12β. Low levels of PTPN6, also known as Src homology region 2 domain-containing phosphatase-1 (SHP-1), are predictor of improved disease-free survival, and could be a useful biomarkers to predict clinical outcome of breast cancer patients.^(28)^ Interestingly, PTPN6 can be self-regulated and is essential for the inhibitory function of NK cells.^(29)^ The cytokine IL-12β seems to be critical in inflammation and adaptive immune responses. High levels of IL-12 predisposed patients to breast cancer.^(30)^ The SIGLEC7 and LAIR1 are directly involved with the inhibition of NK cells, macrophages, T- and B- cells.

It seems that this process involves SIGLEC7 and LAIR1, which are involved in immune evasion that might be modulated by PTPN6 and IL-12β through the PI3K pathway.

### Limitations

A notable limitation of gene correlation analysis is the list of selected genes found in the database, which were pre-determined by previous studies. Moreover, genomics pathway analysis is limited to a known repertoire of genes that have been added to the database. Despite the results showing a correlation between HPSE and other genes, the research was only conducted *in silico*, and therefore, further research *in vivo* or *in vitro* are highly desirable to validate the results. The difference between the number of breast cancer subtype samples varied between luminal A/B (n=321), HER2 (n=58), and triple-negative (n=81).

## CONCLUSION

The signaling pathway analysis using bioinformatics tools gives supporting evidence of possible mechanisms related to breast cancer development. Heparanase-1 expression is co-related with sialic acid-binding immunoglobulin-like lectin 7, leukocyte associated immunoglobulin-like receptor 1, phosphoinositide-3-kinase adaptor protein 1 and cathepsin L. The sialic acid-binding immunoglobulin-like lectin 7, leukocyte-associated immunoglobulin-like receptor 1 and phosphoinositide-3-kinase adaptor protein 1 are directed related with immune system evasion during breast cancer progression. Furthermore, cathepsin L transforms inactive heparanase-1 form into active heparanase-1, both trigger extracellular matrix remodeling, which contributes to enhanced tumor-host interaction of the tumor.

## References

[1] 1. Honts JE. Evolving strategies for the incorporation of bioinformatics within the undergraduate cell biology curriculum. Cell Biol Educ. 2003;2(4):233-47. 10.1187/cbe.03-06-0026 PMC256976 14673489

[2] 2. Sanderson RD, Elkin M, Rapraeger AC, Ilan N, Vlodavsky I. Heparanase regulation of cancer, autophagy and inflammation: new mechanisms and targets for therapy. FEBS J. 2017;284(1):42-55. Review. 10.1111/febs.13932 PMC5226874 27758044

[3] 3. Arvatz G, Weissmann M, Ilan N, Vlodavsky I. Heparanase and cancer progression: New directions, new promises. Hum Vaccin Immunother. 2016; 12(9):2253-6. 10.1080/21645515.2016.1171442 PMC5027699 27054564

[4] 4. Vlodavsky I, Gross-Cohen M, Weissmann M, Ilan N, Sanderson RD. Opposing functions of heparanase-1 and heparanase-2 in Cancer progression. Trends Biochem Sci. 2018;43(1):18-31. Review. 10.1016/j.tibs.2017.10.007 PMC5741533 29162390

[5] 5. Cerami E, Gao J, Dogrusoz U, Gross BE, Sumer SO, Aksoy BA, et al. The cBio cancer genomics portal: an open platform for exploring multidimensional cancer genomics data. Cancer Discov. 2012;2(5):401-4. Erratum in: Cancer Discov. 2012;2(10):960. 10.1158/2159-8290.CD-12-0095 PMC3956037 22588877

[6] 6. Gao J, Aksoy BA, Dogrusoz U, Dresdner G, Gross B, Sumer SO, et al. Integrative analysis of complex cancer genomics and clinical profiles using the cBioPortal. Sci Signal. 2013;6(269):pl1. 10.1126/scisignal.2004088 PMC4160307 23550210

[7] 7. Jellusova J, Rickert RC. The PI3K pathway in B cell metabolism. Crit Rev Biochem Mol Biol. 2016;51(5):359-78. Review. 10.1080/10409238.2016.1215288 PMC5139348 27494162

[8] 8. Wong KA, Wilson J, Russo A, Wang L, Okur MN, Wang X, et al. Intersectin (ITSN) family of scaffolds function as molecular hubs in protein interaction networks. PLoS One. 2012;7(4):e36023. 10.1371/journal.pone.0036023 PMC3338775 22558309

[9] 9. Mace EM. Phosphoinositide-3-Kinase Signaling in Human Natural Killer Cells: New Insights from Primary Immunodeficiency. Front Immunol. 2018;9:445. Review. 10.3389/fimmu.2018.00445 PMC5845875 29563913

[10] 10. Varchetta S, Brunetta E, Roberto A, Mikulak J, Hudspeth KL, Mondelli MU, et al. Engagement of Siglec-7 receptor induces a pro-inflammatory response selectively in monocytes. PLoS One. 2012;7(9):e45821. 10.1371/journal.pone.0045821 PMC3461047 23029261

[11] 11. Angata T, Varki A. Siglec-7: a sialic acid-binding lectin of the immunoglobulin superfamily. Glycobiology. 2000;10(4):431-8. 10.1093/glycob/10.4.431 10764831

[12] 12. Meyaard L. The inhibitory collagen receptor LAIR-1 (CD305). J Leukoc Biol. 2008;83(4):799-803. Review. 10.1189/jlb.0907609 18063695

[13] 13. Sudhan DR, Siemann DW. Cathepsin L targeting in cancer treatment. Pharmacol Ther. 2015;155:105-16. Review. 10.1016/j.pharmthera.2015.08.007 PMC4624022 26299995

[14] 14. Ni M, MacFarlane AW 4th, Toft M, Lowell CA, Campbell KS, Hamerman JA. B-cell adaptor for PI3K (BCAP) negatively regulates Toll-like receptor signaling through activation of PI3K. Proc Natl Acad Sci U S A. 2012;109(1):267-72. 10.1073/pnas.1111957108 PMC3252908 22187458

[15] 15. Vinay DS, Ryan EP, Pawelec G, Talib WH, Stagg J, Elkord E, et al. Immune evasion in cancer: mechanistic basis and therapeutic strategies. Semin Cancer Biol. 2015;35 Suppl:S185-98. Review. 10.1016/j.semcancer.2015.03.004 25818339

[16] 16. Carpentier SJ, Ni M, Duggan JM, James RG, Cookson BT, Hamerman JA. The signaling adaptor BCAP inhibits NLRP3 and NLRC4 inflammasome activation in macrophages through interactions with Flightless-1. Sci Signal. 2019;12(581):eaau0615. 10.1126/scisignal.aau0615 PMC6604799 31088976

[17] 17. Song S, Chew C, Dale BM, Traum D, Peacock J, Yamazaki T, et al. A requirement for the p85 PI3K adapter protein BCAP in the protection of macrophages from apoptosis induced by endoplasmic reticulum stress. J Immunol. 2011;187(2):619-25. 10.4049/jimmunol.0903425 21685326

[18] 18. Kawasaki T, Kawai T. Toll-like receptor signaling pathways. Front Immunol. 2014;5:461. Review. 10.3389/fimmu.2014.00461 PMC4174766 25309543

[19] 19. Macauley MS, Crocker PR, Paulson JC. Siglec-mediated regulation of immune cell function in disease. Nat Rev Immunol. 2014;14(10):653-66. Review. 10.1038/nri3737 PMC4191907 25234143

[20] 20. Rego SL, Helms RS, Dréau D. Tumor necrosis factor-alpha-converting enzyme activities and tumor-associated macrophages in breast cancer. Immunol Res. 2014;58(1):87-100. Review. 10.1007/s12026-013-8434-7 24072428

[21] 21. Wang Y, Zhang X, Miao F, Cao Y, Xue J, Cao Q, et al. Clinical significance of leukocyte-associated immunoglobulin-like receptor-1 expression in human cervical cancer. Exp Ther Med. 2016;12(6):3699-705. 10.3892/etm.2016.3842 PMC5228450 28105100

[22] 22. Saverino D, Fabbi M, Merlo A, Ravera G, Grossi CE, Ciccone E. Surface density expression of the leukocyte-associated Ig-like receptor-1 is directly related to inhibition of human T-cell functions. Hum Immunol. 2002;63(7):534-46. 10.1016/s0198-8859(02)00409-3 12072189

[23] 23. Zhang W, Yang HC, Wang Q, Yang ZJ, Chen H, Wang SM, et al. Clinical value of combined detection of serum matrix metalloproteinase-9, heparanase, and cathepsin for determining ovarian cancer invasion and metastasis. Anticancer Res. 2011;31(10):3423-8. 21965756

[24] 24. Meyaard L, Hurenkamp J, Clevers H, Lanier LL, Phillips JH. Leukocyte-associated Ig-like receptor-1 functions as an inhibitory receptor on cytotoxic T cells. J Immunol. 1999;162(10):5800-4. 10229813

[25] 25. Choong PF, Nadesapillai AP. Urokinase plasminogen activator system: a multifunctional role in tumor progression and metastasis. Clin Orthop Relat Res. 2003(415 Suppl):S46-58. Review. 10.1097/01.blo.0000093845.72468.bd 14600592

[26] 26. Zeng Y, Yao X, Chen L, Yan Z, Liu J, Zhang Y, et al. Sphingosine-1-phosphate induced epithelial-mesenchymal transition of hepatocellular carcinoma via an MMP-7/ syndecan-1/TGF-β autocrine loop. Oncotarget. 2016;7(39):63324-37. 10.18632/oncotarget.11450 PMC5325366 27556509

[27] 27. Vlodavsky I, Beckhove P, Lerner I, Pisano C, Meirovitz A, Ilan N, et al. Significance of heparanase in cancer and inflammation. Cancer Microenviron. 2012;5(2):115-32. 10.1007/s12307-011-0082-7 PMC3399068 21811836

[28] 28. Youssef G, Gillett C, Agbaje O, Crompton T, Montano X. Phosphorylation of NTRK1 at Y674/Y675 induced by TP53-dependent repression of PTPN6 expression: a potential novel prognostic marker for breast cancer. Mod Pathol. 2014;27(3):361-74. 10.1038/modpathol.2013.129 23948750

[29] 29. Viant C, Fenis A, Chicanne G, Payrastre B, Ugolini S, Vivier E. SHP-1-mediated inhibitory signals promote responsiveness and anti-tumour functions of natural killer cells. Nat Commun. 2014;5:5108. 10.1038/ncomms6108 25355530

[30] 30. Kaarvatn MH, Vrbanec J, Kulic A, Knezevic J, Petricevic B, Balen S, et al. Single nucleotide polymorphism in the interleukin 12B gene is associated with risk for breast cancer development. Scand J Immunol. 2012;76(3):329-35. 10.1111/j.1365-3083.2012.02736.x 22702905

