# Supplementary material for: In silico investigation of heparanase-correlated genes in breast cancer subtypes
Source: Einstein (Sao Paulo). 2020 Oct 2;18:eAO5447. doi: 10.31744/einstein_journal/2020AO5447 (PMC7531901; doi:10.31744/einstein_journal/2020AO5447)
Supplement: Translation

# Translation: Investigação *in silico* dos genes correlacionados à heparanase nos diferentes subtipos de câncer de mama

**Authors:** Carina Mucciolo Melo, Henrique Pereira Prado, Gabriela Araújo Attie, Daniel Lacaz Ruiz, Manoel João Batista Castello Girão, Maria Aparecida da Silva Pinhal

**Affiliations:** 1 Brasil Faculdade de Medicina do ABC, Santo André, SP, Brasil.; 2 Brasil Universidade Federal de São Paulo, São Paulo, SP, Brasil.; 3 Canada McMaster University, Hamilton, ON, Canada.

**Keywords:** Heparanase, Biologia computacional, Neoplasias da mama

## Abstract

**Objetivo:**

Investigar os genes que podem estar relacionados aos mecanismos que modulam a heparanase-1.

**Métodos:**

A análise foi realizada na Universidade Federal de São Paulo, utilizando dados fornecidos por: *The Cancer Genome Atlas*, *University of California Santa Cruz Genome Browser*, *Kyoto Encyclopedia of Genes and Genomes Pathway Database*, *Database for Annotation*, *Visualization and Integrated Discovery Bioinformatics Database* e os softwares cBioPortal e *Ingenuity Pathway Analysis.*

**Resultados:**

Usando o perfil de expressão de RNA mensageiro de diferentes subtipos moleculares de câncer de mama, propusemos que a heparanase-1 esteve correlacionada com a progressão tumoral. Além disso, os genes analisados apresentaram coexpressão com heparanase-1. Os resultados mostraram que a heparanase-1 coexpressa com proteína adaptadora 1 da fosfoinositídeo 3-quinase, lectina 7 tipo Ig de ligação ao ácido siálico e receptor 1 do tipo imunoglobulina associado a leucócitos, estes genes estão diretamente relacionados à evasão do sistema imune durante a progressão do câncer de mama. Além disso, a catepsina L foi coexpressa com a heparanase-1 e transformou a forma inativa da heparanase-1 em heparanase-1 ativa, desencadeando o remodelamento da matriz extracelular, o que contribuiu para a interação do tumor com o ambiente tumoral.

**Conclusão:**

A análise utilizando bioinformática fornece evidências de possíveis mecanismos relacionados ao desenvolvimento do câncer de mama. Genes de evasão do sistema imune foram coexpressos com a heparanase-1, uma enzima relacionada à progressão tumoral.

## INTRODUÇÃO

A publicação do primeiro texto do genoma humano em 2001 marcou o início de uma nova era na biologia: a era da bioinformática. Dada a enorme quantidade de informações que estão sendo digitalizadas, os dados genômicos só podem ser claramente entendidos com a ajuda de ferramentas de ciência de dados e bioinformática, como programas de análise de vias. Uma vez que a quantidade dessas bases de dados cresce exponencialmente, a dependência dos profissionais de saúde e pesquisadores em relação a essas ferramentas computacionais parece estar sempre aumentando.^(1)^ Embora a análise de dados em bases virtuais amplamente disponíveis seja um recurso útil, a acessibilidade ao uso de dados de bioinformática não é amplamente divulgada em toda a comunidade científica, devido à *expertise* necessária para usar os bancos de dados.

Um dos objetivos deste estudo é fornecer documentação norteadora para os profissionais de saúde sobre como responder a questões biológicas relacionadas à expressão genética específica e sua correlação com outras vias de sinalização.

Propusemos realizar este estudo em ambas as isoformas da heparanase – a heparanase-1 (HPSE) e a heparanase-2 (HPSE2) –, no contexto do câncer de mama.

A HPSE é uma endo-beta-glucuronidase que participa na clivagem das cadeias do sulfato de heparano e da heparina. Os oligossacarídeos gerados pela ação enzimática da HPSE interagem com citocinas, fatores angiogênicos e fatores de crescimento, ativando processos relacionados à progressão do tumor. Além disso, estudos recentes mostraram a correlação da HPSE com a formação de exossomos, a ativação do sistema imune, a autofagia e a quimiorresistência, demonstrando sua importância na modulação da ativação cruzada (*crosstalk*) entre as células tumorais e o ambiente tumoral. Como resultado desse achado, a HPSE é um alvo para o desenvolvimento de novos medicamentos para o câncer. O tratamento anti-HPSE pode ser usado para estimular as células T e iniciar respostas imunes anticâncer.^(2,3)^

Ao contrário da HPSE, a HPSE2 possui propriedade catalítica, e sua função ainda é desconhecida. No entanto, sabe-se que a HPSE2 interage com heparina e com sulfato de heparano, podendo ser usada para dificultar os efeitos da HPSE.^(4)^

Apesar da importância da HPSE e sua contribuição para a carcinogênese, a angiogênese e a inflamação, há uma quantidade limitada de trabalhos que detalham como outros genes podem ser correlacionados com os mecanismos de ação da HPSE. Para responder a essa pergunta, usamos como fontes de análise bases de dados genômicos disponíveis gratuitamente online.

Também a correlação de HPSE e HPSE2 em diferentes tipos de câncer de mama humano foi abordada para responder se ambas as heparanases poderiam ser um marcador útil para diferenciar esses subtipos de câncer.

## OBJETIVO

Investigar os genes que podem estar relacionados aos mecanismos que modulam a heparanase-1.

## MÉTODOS

### Bases de dados genômicos

Uma análise foi conduzida na Universidade Federal de São Paulo, em São Paulo (SP), em dados fornecidos pelas bases *The Cancer Genome Atlas* (TCGA), *University of California Santa Cruz* (UCSC) *Genome Browser, Kyoto Encyclopedia of Genes and Genomes* (KEGG) *Pathway Database, Database for Annotation, Visualization and Integrated Discovery* (DAVID) e *Bioinformatics Database*. As análises foram realizadas de 2017 até 2019. Este estudo não necessitou de aprovação do Comitê de Ética.

### Serviços em nuvem

Os serviços em nuvem via internet foram utilizados para analisar as bases de dados genômicos: um aplicativo baseado na internet cBioPortal, para análise da base de dados TCGA.

### Coleta de dados da coorte

Foram coletados dados de pacientes com câncer de mama como parte do projeto do TCGA sobre carcinoma invasivo de mama e classificados de acordo com o subtipo (luminal A, luminal B, receptor tipo 2 do fator de crescimento epidérmico humano – HER2 – positivo e triplo-negativo).

### Análise de correlação da heparanase e genes coexpressos

Serviços em nuvem do cBioPortal relacionados às bases de dados genômicos foram utilizados para analisar a expressão gênica da coorte selecionada.

### Análise das vias de sinalização

As vias de genes coexpressos com as isoformas de HPSE foram analisadas utilizando o software de vias de transdução de sinal *Ingenuity Pathway Analysis* (IPA).

### Teste de significância estatística

O software (SPSS Inc^®^, Illinois, USA), versão 17.0, foi usado para analisar o conjunto de dados fornecido pelas bases de dados genômicos, utilizando um limiar de significância estatística de 5% (p≤0,05). Todos os conjuntos de dados foram importados após serem reorganizados em tabelas. O teste Kolmogorov-Smirnov e o teste *t* de Student foram aplicados por este software.

## RESULTADOS

Primeiramente, analisamos os dados baixados de www.cbioportal.org e pertinentes ao câncer de mama invasivo (TCGA, Nature 2012).^(5,6)^ Os dados coletados referem-se à expressão de RNA mensageiro (mRNA) para HPSE e HPSE2 em diferentes subtipos de câncer de mama, como luminal A/B (n=321), triplo-negativo/basal (n=81), e HER2 (n=58) (Figura 1).

**Figura 1:** Expressão do RNA mensageiro da heparanase-1 e da heparanase-2 em diferentes subtipos de câncer de mama. Expressão relativa do RNA mensageiro da (A) heparanase-1 e (B) heparanase-2. Análise foi realizada nos subtipos: câncer de mama luminal A/B (n=321), com receptor tipo 2 do fator de crescimento epidérmico humano amplificado (n=58) e basal/triplo-negativo (n=81). Os dados foram obtidos na base de dados do *The Cancer Genome Atlas Breast Carcinoma* (2012) mRNA: RNA mensageiro; HPSE: heparanase-1; HER2: receptor tipo 2 do fator de crescimento epidérmico humano; HPSE2: heparanase-2.

Houve diferença estatisticamente significativa entre as expressões de HPSE em vários subtipos de câncer de mama. No entanto, nenhuma diferença foi detectada para a HPSE2.

Considerando-se que a HPSE2 não mostrou correlação significativa entre os subtipos de tumor, usamos a HPSE para avaliar genes coexpressos. A análise da base de dados TCGA mostrou genes que se correlacionavam positivamente com a expressão de HPSE. Isso incluiu a proteína adaptadora 1 da fosfoinositídeo-3-quinase (PIK3AP1), as lectinas semelhantes à imunoglobulina de ligação ao ácido siálico (SIGLEC7), o receptor 1 imunoglobulina-like associado a leucócitos (LAIR1), e a catepsina L (CTSL).

A PIK3AP1 desempenha múltiplas funções na resposta imune e tem papel crucial como componente a jusante de vários receptores relacionados à imunidade, em muitos tipos de células. Nas células B, PIK3AP1 liga o receptor de células B à subunidade p85 da fosfoinositídeo-3-quinase (PI3K), sendo responsável pela maturação celular, enquanto nas células *natural killer* (NK), essa proteína atua como mediadora entre os receptores inibidores de NK e PI3K/proteína quinase B alfa (AKT).^(7-9)^

A figura 2 mostra correlação significativa entre o aumento da expressão de HPSE e a expressão de PIK3AP1 nos subtipos de tumor A/B luminal e triplo-negativo. No entanto, não observamos correlação significativa entre HPSE e PIK3AP1 em tumores com HER2 amplificado, indicados por um coeficiente R^2^ não significante e valor de p.

**Figura 2:** Correlação entre heparanase-1 e proteína adaptadora 1 da fosfoinositídeo-3-cinase em diferentes subtipos de câncer de mama: (A) luminal A/B (n=321), (B) amplificado (n=58) e (C) triplo-negativo (n=81) PIK3AP1: proteína adaptadora 1 da fosfoinositídeo-3-cinase; HPSE: heparanase-1.

As SIGLEC7 são receptores inibitórios encontrados nas células NK. Uma vez ligados ao ácido siálico, as SIGLEC7 causam uma cascata a jusante, que impede a degranulação das células NK.^(10)^ Foi observada correlação positiva significativa entre HPSE e SIGLEC7 no câncer de mama luminal A/B, com HER2 amplificado e triplo-negativo (y=0,55x-0,24; R^2^=0.51; p=3,06 x 10^-76^; n=460; triplo-negativo, y=0,1191x+0,5550; R^2^=0,52; p=0,0003; HER2 amplificado, y=0,1387x+0,6429; R^2^=0,6509; p<0,0001; Luminal, y=0,003224x+0,4915; R^2^ =0,5019; p<0,0001) (Figura 3A). É importante enfatizar que as células tumorais superexpressam ligantes sializados, o que resulta em uma inibição aumentada das respostas imunes.^(11)^

**Figura 3:** Coexpressão de RNA mensageiro com heparanase-1. (A) Expressão de lectinas semelhantes à imunoglobulina de ligação ao ácido siálico em relação à expressão do RNA mensageiro da heparanase-1. Os pontos de dados indicam paciente com câncer de mama. A linha de regressão é modelada por y=0,55x – 0,24, R2=0,51, p=3,06 x 10-76, n=460. (B) Expressão do RNA mensageiro do receptor imunoglobulina-like associado a leucócitos, em relação à expressão do RNA mensageiro da heparanase-1. Os pontos de dados indicam paciente com carcinoma de mama. A linha de regressão é modelada por y=0,66x + 0,79, R2=0,46, p=1,24 x 10-63, n=460. (C) Expressão do RNA mensageiro catepsina L em relação à expressão do RNA mensageiro da heparanase-1. Os pontos de dados indicam pacientes com câncer de mama. A linha de regressão é modelada por y=0,58x – 0,69, R2=0,48, p=6,17 x 10-65, n=460 SIGLEC7: lectinas semelhantes à imunoglobulina de ligação ao ácido siálico; HPSE: heparanase-1; mRNA: RNA mensageiro; LAIR1: receptor 1 imunoglobulina-like associado a leucócitos; CTSL: catepsina L.

O LAIR1 inibidor é uma glicoproteína transmembrana, que, por sua vez, é um receptor do colágeno, o qual promove a supressão das respostas imunes. O LAIR1 é expresso na maioria das células imunes, incluindo células NK, linfócitos T e B, eosinófilos e basófilos.^(12)^

Houve correlação direta entre a expressão de LAIR1 e HPSE (y=0,66x+0,79; R^2^=0,46; p=1,24 x 10^-63^; n=460; triplo-negativo, y=0,5386x-0,08710; R^2^=0,5204; p=0,0014; HER2 amplificado; y=0,6002x+0,09642; R^2^=0,5918; p=0,0003; Luminal, y=0,4148x+0,02971; R^2^=0,4061; p<0.0001), como mostrado na figura 3B.

A CTSL é uma protease da cisteína,^(13)^ que mostra correlação positiva com HPSE em três subtipos de carcinoma da mama, luminal A/B, com HER2 amplificado, e triplo-negativo (y=0,58x-0,69; R^2^=0,48, p=6,17 x 10^-65^, n=460; basal subtype, y=0,4291x-0,005506; R^2^=0,4691; p=0,0034; HER2-enriched, y=0,3187x-0,1288, R^2^=0,3480; p=0,0437; Luminal, y=0,3936x+0,003278, R^2^ =0,4144, p<0,0001 ) (Figura 3C).

A análise da base de dados TCGA mostrou que a expressão dos genes HPSE, PIK3AP1, SIGLEC7, LAIR1 e CTSL tem correlações positivas de acordo com os subtipos de câncer de mama. Enquanto HPSE e CTSL estão envolvidos na remodelação da matriz extracelular (MEC), PIK3AP1, SIGLEC7 e LAIR1 fazem parte das vias de sinalização envolvidas na inibição do sistema imune.^(12-15)^

## DISCUSSÃO

Nos últimos anos, tem sido grande o progresso para esclarecer alguns mecanismos de evasão imunológica e carcinogênese. Os conceitos básicos desse processo envolvem células imunes, que apresentam receptores capazes de evitar uma exacerbação inflamatória, a qual ocorre durante o desenvolvimento do tumor. Os mesmos mecanismos que previnem doenças autoimunes também podem ser utilizados para diminuir as respostas antitumorais.^(15)^

A via de sinalização mediada por PI3K promove a fosforilação de AKT, resultando em um evento crucial na tumorigênese. A AKT ativa promove a localização do fator de transcrição FOXO O1, beta-catenina, entre outros, para o citoplasma, modulando mecanismos relacionados à proliferação celular, à apoptose e à metástase.^(14-17)^

O adaptador de célula B para PIK3AP1, também chamado BCAP, é uma proteína de sinalização que ativa a via PI3K a jusante da sinalização dos receptores celulares em células B e de receptor tipo Toll (TLR) em macrófagos. A PIK3AP1 se liga à subunidade p85 reguladora da classe I PI3K e é uma grande proteína de múltiplos domínios.^(16)^

A PIK3AP1 está envolvida em múltiplas respostas dos receptores e confere respostas variadas, dependendo do tipo de célula. A expressão de PIK3AP1 envolve a ativação de TLR que possuem importantes funções no processo inflamatório, na regulação da resposta imune e na proliferação celular. Os TLR ativados promovem a ativação da via PIK3AP1, que leva à produção de fator nuclear kappa B (NF-κB), regulando a expressão de citocinas por meio de vários complexos, incluindo MyD88, TIRAP/Mai e TRF. A ativação da via NF-κB solicita uma resposta do sistema imune adaptativo, pela produção de citocinas inflamatórias, como interleucina (IL) 1, IL-8 e fator de necrose tumoral alfa (TNF-α), entre outros fatores pró-inflamatórios, principalmente nas células circulantes, como as NK e os macrófagos. O NF-κB tem papel fundamental na regulação da resposta imune durante uma infecção bacteriana ou viral. A regulação incorreta do NF-κB leva ao desenvolvimento de tumores e de doenças inflamatórias e autoimunes.^(14,17,18)^

O receptor SIGLEC7, presente nas células NK e macrófagos, liga-se a conjugados de glicol alternados na superfície das células tumorais. Essa interação impede que as células NK provoquem a morte de células tumorais por desgranulação.^(19)^ É interessante notar que foi observada expressão aumentada de SIGLEC7 em macrófagos que residem no microambiente tumoral.^(19,20)^ As SIGLEC7 presentes em monócitos induzem a produção de fatores inflamatórios, como TNF-α, IL-1α, IL-6, IL-8, MIP-1β.^(10)^ Além disso, patógenos e células tumorais que superexpressam AS e ligantes de carboidratos de SIGLEC7 contribuem para a inibição de células NK e linfócitos T. Portanto, as SIGLEC7 parecem estar relacionadas ao escape tumoral.^(19)^

O LAIR1 interage com o colágeno encontrado na MEC dos tumores, inibindo a citotoxicidade de células NK e linfócitos T. Além disso, os tecidos obtidos do câncer do colo do útero humano apresentam maior expressão em comparação com os tecidos normais, como verificado na expressão da HPSE.^(12,21,22)^

A CTSL cliva vários componentes da MEC, como fibronectina, colágeno e laminina. Sabe-se também que a CTSL processa a pró-HPSE (inativa) em HPSE (ativa), por meio de um mecanismo proteolítico, sendo que ambos os complexos proteicos são secretados para o meio extracelular. A superexpressão dessas enzimas é vista em vários tipos de tumores, como glioma, melanoma e câncer de fígado, mama e próstata. A HPSE e a CTSL são fatores importantes no remodelamento da MEC.^(13)^ Zhang et al., verificaram que a CTSL e a HPSE estavam aumentadas no câncer de ovário, e ambas estavam correlacionadas à progressão do tumor.^(23)^

A CTSL parece ter papéis importantes na tumorigênese tanto no núcleo quanto na MEC. A CTSL secretada é capaz de degradar componentes da MEC, como laminina, colágeno tipo I e IV, fibronectina, elastina e E-caderina, desempenhando papel importante na remodelação da MEC. Além disso, a CTSL participa da ativação proteolítica do plasminogênio da uroquinase e da catepsina D, que atuam como fatores tumorigênicos.^(13,24,25)^ A CTSL no núcleo é responsável pelo processamento do Cux p200, que leva à produção de genes mesenquimais. Também degrada o S38P1, que ativa o reparo do DNA.^(13)^

Outros fatores que podem afetar o microambiente tumoral são hipóxia e acidose, que contribuem para a secreção de CTSL a partir do lisossomo, facilitando as metástases tumorais e aumentando a degradação proteolítica da MEC e da membrana basal.^(13)^

A figura 4 mostra possíveis interações proteicas entre HPSE, PIK3AP1, SIGLEC7, LAIR1 e CTSL, que inferimos da análise de vias usando o software IPA. PI3KAP1 e CTSL são diretamente relacionados à HPSE. As vias de sinalização da PI3K/AKT acionam a expressão de HPSE e induzem transição de epitelial a mesenquimal, sinalizando metástases de hepatocarcinoma.^(26)^ A CTSL está envolvida na ativação da HPSE e ambas as proteínas participam ativamente no processo de desenvolvimento do tumor.^(27)^ Além disso, LAIR1 e SIGLEC7 se correlacionam com HPSE, e a interação entre SIGLEC7 e HPSE parece ser mediada por proteína tirosina fosfatases não receptoras tipo 6 (PTPN6) e IL-12β. Baixos níveis de PTPN6, também conhecida como região de fosfatase 1 com domínio contendo homologia da região Src 2 (SHP-1), são preditores de melhora da sobrevida livre de doença e podem ser biomarcadores úteis para predizer o desfecho clínico de pacientes com câncer de mama.^(28)^ Curiosamente, a PTPN6 pode ser autorregulada e é essencial para a função inibitória das células NK.^(29)^ A citocina IL-12β parece ser crucial na inflamação e nas respostas imunes adaptativas. Observou-se que altos níveis de IL-12 predispunham as pacientes ao câncer de mama.^(30)^ A SIGLEC7 e o LAIR1 estão diretamente envolvidos na inibição da células NK, macrófagos, células T e B.

**Figura 4:** Representação esquemática da relação entre heparanase, proteína adaptadora 1 da fosfoinositídeo-3-quinase, catepsina L, lectinas semelhantes à imunoglobulina de ligação ao ácido siálico e receptor 1 imunoglobulina-like associado a leucócitos HPSE: heparanase; PIK3AP1: proteína adaptadora 1 da fosfoinositídeo-3-quinase; CTSL: catepsina L; SIGLEC7: lectinas semelhantes à imunoglobulina de ligação ao ácido siálico; LAIR1: receptor 1 imunoglobulina-like associado a leucócitos.

Aparentemente, esse processo envolve as SIGLEC7 e o LAIR1, que participam da evasão imune, que pode ser modulada por PTPN6 e IL-12β por meio da via PI3K.

### Limitações

Uma limitação importante da análise da correlação genética é que os genes selecionados foram encontrados em base de dados, logo a lista de genes é predeterminado. Além disso, a análise das vias genômicas é limitada a um repertório de genes conhecidos, que foram adicionados à base de dados. Apesar de os resultados mostrarem correlação entre HPSE e outros genes, a pesquisa somente foi conduzida *in silico*. Pesquisas futuras *in vivo* or *in vitro* são altamente desejáveis para validar os resultados. A diferença entre o número de amostras de subtipos de câncer de mama varia entre luminal A/B (n=321), HER2 (n=58) e triplo-negativo (n=81).

## CONCLUSÃO

A análise da via de sinalização utilizando ferramentas de bioinformática fornece evidências que apoiam possíveis mecanismos relacionados ao desenvolvimento do câncer de mama. A expressão da heparanase-1 está correlacionada com lectinas semelhantes à imunoglobulina de ligação ao ácido siálico, receptor 1 imunoglobulina-like associado a leucócitos, proteína adaptadora 1 da fosfoinositídeo-3-quinase e catepsina L. Os três primeiros são diretamente relacionados à evasão do sistema imune durante a progressão do câncer de mama. Já a catepsina L transforma a forma inativa de heparanase-1 em heparanase-1 ativa, ambas desencadeando o remodelamento da matriz extracelular e contribuindo para intensificar a interação tumor-hospedeiro do tumor.
